# Prevalence of Dietary Supplement Use in Patients with Proven or Suspected Cardiovascular Disease

**DOI:** 10.1155/2011/632829

**Published:** 2010-10-13

**Authors:** Yu Sun Bin, Hosen Kiat

**Affiliations:** ^1^Cardiac Health Institute, 173 Shaftsbury Road, Eastwood, NSW 2122, Australia; ^2^School of Advanced Medicine, Macquarie University, NSW 2109, Australia

## Abstract

Systematic search of bibliographic databases was conducted to describe the prevalence of dietary supplement use in cardiac patients. Included for review were studies that investigated supplement use in people with cardiovascular risk factors or proven cardiovascular disease. Databases searched were Medline, EMBASE, CINAHL, AMED, Meditext, H&S and IPA. Over five hundred articles were retrieved and twenty studies met the criteria for this review. Dietary supplements were taken by a median 36% (interquartile range: 26–42%) of cardiac patients; 36% (IQR 18–43%) reported taking a vitamin/mineral supplement and 12% (IQR 7–21%) used herbal supplements. Many users indicated that supplements were taken specifically for heart health and 16–64% of users reported using supplements alongside prescription medications. However 39–95% of treating physicians were unaware of patients' supplement use. Dietary supplement use in patients with cardiovascular disease appears common, as does the concurrent use of supplements with prescription medicines. This information is often not communicated to doctors and treating physicians may need to be more proactive in asking about supplement use.

## 1. Introduction

A dietary supplement is defined as “any product intended for ingestion as a supplement to the diet” [1]. These substances include amino acids, charcoal, choline salts, essential oils, plant or herbal material, homeopathic preparations, nonvaccine microorganisms, minerals, nonhuman animal material, lipids, substances from bees, and vitamins or provitamins. In this paper, we use the term “dietary supplement” to indicate any of these substances used as complementary and alternative medicine (CAM) and taken orally for the improvement of health or the prevention of illness. 

The use of dietary supplements is common; in the general population, dietary supplements are the second most commonly used type of CAM after prayer [2]. Dietary supplements can interfere with the biotherapeutic action of prescription medications, and this is of particular concern in cardiac patients, many of whom are on long-term medications and are at increased risk of acute life-threatening events. Chronic conditions such as arthritis, cancer, depression, and anxiety have been consistently associated with CAM use [3], so it is probable that patients with chronic cardiovascular disease are also likely to use CAM. We conducted a systematic review of the literature to find the prevalence of dietary supplement use by cardiac patients and to identify commonly used supplements. 

## 2. Method

### 2.1. Study Selection

A literature search was carried out on 2 December 2009. We searched the following databases from their earliest availability up to and including November 2009: Medline through OvidSP from 1950, EMBASE through embase.com from 1980, CINAHL through EBSCO Host from 1982, Allied and Complementary Medicine (AMED) through OvidSP from 1985, Australian Medical Index (Meditext) through Informit from 1968, Health & Society (H&S) through Informit from 1980, and International Pharmacy Abstracts (IPA) through OvidSP from 1970. 

MeSH keywords and qualifiers were used for databases indexed using this method, that is, Medline and CINAHL. We used the MeSH terms “cardiovascular diseases” with the qualifier “/therapy” to find articles relevant to treatment of cardiovascular disease. To find articles on dietary supplement use, we used the MeSH terms “medicine, traditional”, “dietary supplements”, “vitamins”, “minerals”, each qualified with “/utilization”. All MeSH terms were exploded to include subheadings. The “cardiovascular disease” search results were then intersected with each of the dietary supplement searches. For databases not indexed using MeSH, searches were performed using the same terms without the qualifiers. In these databases, additional searches were conducted using the terms “cardiovascular diseases”, “cardiology”, and “cardiac” intersected with each of “complementary and alternative medicine”, “dietary supplement”, and “herbal medicine”. Search results were limited to articles in English and those that dealt with humans where these options were available. 

**Figure 1:**
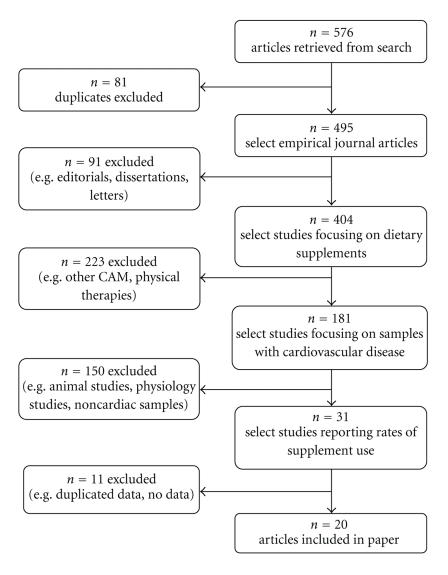
Flowchart showing the selection of studies from search results.

The titles and abstracts of articles were read and refined to include only those studies that (i) reported the prevalence of dietary supplement use, (ii) involved a sample of patients either attending for cardiac care or those reporting a cardiovascular condition, and (iii) indicated the types of supplements used. If abstracts were not available or were ambiguous with regard to these criteria, the full-text article was obtained for inspection. Articles were excluded if (i) study methods were not described, (ii) prevalence data on dietary supplement use were not reported (or these could not be calculated from reported data), and if (iii) they were not in English. Citations in the relevant papers were also used to locate articles not found by the above methods.  Figure 1 shows the selection of studies from the search results. 

### 2.2. Data Extraction

Two data tables were designed for systematic extraction of study information. Methodological details were extracted and compared in Table 1, while study content and results were extracted and summarised in Table 2. The most common supplements are listed in Table 3, and for the sake of brevity, this only includes supplements used by ≥1% of patients and which were reported in 2 or more of the included studies. 

### 2.3. Statistical Analyses

Prevalence of overall supplement use was obtained from each study. For studies that did not report the overall prevalence, the figure corresponding to the most common supplement or most common supplement category was used. This method is expected to underestimate rather than overestimate the total number of supplement users. The median prevalence across studies and the corresponding interquartile ranges (IQR) were calculated for (i) any supplement use, (ii) the use of vitamin/mineral supplements, and (iii) the use of herbal supplements. Where studies reported use of individual supplements, data was extracted and pooled figures are presented in Table 3. 

## 3. Results

### 3.1. Study Characteristics

Of the 576 studies found, 22 studies met the inclusion criteria. Two of these were excluded [4, 5] because they analysed subsets of the same data as another study [6]. Therefore, 20 studies were included for review [6–25]. 

All included studies were conducted between 1995 and 2008. Eleven were from the United States [6, 8, 9, 11–14, 17, 18, 21, 25], three were from Canada [7, 19, 23] and the others were from Hong Kong [22], India [20], Italy [15], Nigeria [10], Turkey [24], and the United Kingdom [16]. Three studies analysed data from population surveys in the US [6, 11, 13], one contacted a registry of patients with cardiovascular disease [23], and the others sampled consecutive patients by convenience from their respective hospital, outpatient, or specialist cardiology clinic [7–10, 12, 14–22, 24, 25]. Of the studies involving clinical samples, five collected data by way of a patient-completed survey [7, 17–19, 25], seven conducted face-to-face interviews [10, 12, 14–16, 20, 24], two used a combination of telephone and face-to-face interviews [8, 9], and two drew on information from routine clinical examination [21, 22]. 

Thoroughness of methodological reporting varied between studies (Table 1). Only three of the 16 clinical studies commented on the representativeness of their sample compared to the greater clinical population [7, 19, 23]. Six did not report the survey response rate [9, 10, 14, 19, 21, 24]. For those that did, responding ranged from 26%–100% (mean 74%). Only four of the clinical studies piloted their survey instrument [7, 19, 24] and two trained multiple interviewers to an equivalent standard [12, 23]. Four performed a sample-size calculation to determine whether there was sufficient statistical power to detect differences between supplement users and nonusers [8, 14, 23, 25]. Many studies did not explicitly state what was meant by “dietary supplement” or CAM and three studies did not define the time period over which supplement use was surveyed [9, 10, 23]. 

Six out of 20 studies surveyed the use of CAM as well as dietary supplements [6, 8, 10, 12, 20, 23], which included physical therapies such as chiropractic and yoga, and mind-body therapies such as relaxation and hypnosis. Another four included the use of nonprescription medications as well as dietary supplements [7, 9, 11, 19]. Only results specific to dietary supplements are reported in this paper. 

### 3.2. Prevalence of Dietary Supplement Use

Any supplement use was reported by a median 36% (IQR 26%–42%) of patients across all 20 studies. Vitamins and minerals were taken by a median 36% (IQR 18%–43%) of patients across 17 studies. Herbal substances were taken by a median 12% (IQR 7%–21%) of patients across all 20 studies. Other supplements (fish oil, glucosamine, homeopathy, etc.) were taken by a median 7% of patients (IQR 5%–10%) across 14 studies.

We also analysed results with respect to time periods surveyed. Seven studies looked at ever or yearly use, finding a median 25% of respondents used supplements (IQR 17%–45%). Three or 6-month use was surveyed by four studies with median 49% users (IQR 37%–61%). Median use within the last month in six studies was found to be 39% (IQR 37%–40%). Only two studies investigated the duration of supplement use, which averaged 39 months [24], with 88% of herbal users taking the supplement for a month or more [13]. 

Supplement users tended to use more than one supplement at a time: two studies reported an average of 2 supplements per user [14, 24], while another recorded an average of 3 supplements per user [21]. Common supplements used by cardiac patients are summarized in Table 3 with the most common by far being multivitamins and minerals. 

### 3.3. Concomitant Use of Supplements and Prescription Medications

Five studies reported data on the concomitant use of prescription medication and dietary supplements [11, 13, 17, 20, 22, 23]. The study of Indian patients indicated 64% were using CAM in conjunction with prescription antihypertensives, although 86% had started conventional treatment first, while 9% had began CAM first and 5% had started both alternative and prescription treatments simultaneously [20]. One population survey in the US indicated that 36% of vitamin/mineral users and 16% of other supplement users used prescription medication for cardiovascular health [11], while another found that 63% of those with hypertension and 64% of those with high cholesterol were found to also take dietary supplements with their medications for cardiovascular disease [13]. Thirteen percent of Canadian patients with diagnosed coronary artery disease (CAD) had used a dietary supplement in conjunction with warfarin, amiodarone, sotalol, or digoxin [23], while 26% of Chinese patients on warfarin had taken a herbal medicine in the previous week [22]. Supplement users were as likely as nonusers to be taking aspirin, betablockers, ACE inhibitors, statins, and warfarin [17]. There was no difference between herb users and nonusers in terms of indication for warfarin, and the duration and dosage of warfarin therapy [22]. 

Three studies looked at the impact of supplement use on compliance [10, 15, 16]. Of the two which surveyed hypertensive patients [10, 16], one found no difference between supplement users and nonusers on adherence to antihypertensive medication [10], while the other found that female supplement users had lower adherence to prescription medications [16]. The third study that investigated outpatients with heart failure reported that 1 of 22 herbal supplement users reduced or interrupted heart failure medications while taking herbal medicine [15]. 

### 3.4. Cardiovascular Health and Supplement Use

In patients with heart failure, up to 82% of supplement users were taking a supplement specifically for cardiovascular health [25]. In patients with diagnosed cardiovascular disease, 18 out of 42 supplements (43%) were taken for a cardiac reason [14]. 

Supplement users were more likely than nonusers to be under specialist cardiology care [9], although the use of herbal supplements was not associated with previous myocardial infarction or previous percutaneous coronary intervention in a Turkish sample [24]. A US study [21] did find higher supplement use in those with significant CAD (defined as prior myocardial infarction or previous percutaneous coronary intervention), and the studies of Ai and Bolling [8, 26] showed that use of herbs and folk remedies were significantly associated with arrhythmia and left main disease. Six studies specifically investigated patients with congestive heart failure (CHF) [7, 14, 15, 19, 23, 25]. In this population, supplement use was reported to be 40% [23] and 82% [25] although use appeared unrelated to the severity of CHF [7] or left ventricular ejection fraction [9, 21]. 

Four studies reported data on measurable outcomes. In Chinese patients taking warfarin, INR was significantly lower in herbal users than nonusers [22]. In two separate studies [10, 21] of clinic outpatients, blood pressure control did not differ between supplement users and nonusers although herbal use was associated with lower blood pressure in elderly patients with heart failure [9]. In one clinic population, low-density lipoprotein (LDL) levels also did not differ between supplement users and nonusers but high-density lipoprotein (HDL) was higher among the supplement users [21]. 

### 3.5. Patient Perceptions of Dietary Supplements

Seven studies investigated patient perceptions of dietary supplements [6, 9, 14, 17, 20, 23, 24]. The most common reasons for supplement use were: to promote or maintain health (up to 47%), for overall wellbeing (up to 42%), and for energy (up to 25%) [9]. Dissatisfaction with conventional treatment was cited by 17% of patients in one study as a reason for supplement use [23] while in another, 59% cited fear of adverse drug reactions as the reason for using CAM [20]. Three times more supplement users than nonusers thought supplements were safe (75% versus 26%) [14], with 45% of users believing supplements were safer than prescription medications [15], 30% indicating that supplements were less likely to interact with other medicines [17], and 45%–47% thinking that supplements would result in fewer side effects compared to prescription medications [14, 17]. Two studies found that 47% [17] and 81% [24] of users, respectively, were unaware herbal medicines could impact negatively on prescription medications. Dietary supplements were viewed as effective by 70%–80% of supplement users [6, 14]. 

Only one study uncovered negative views of CAM [20], finding that 56% of patients were dissatisfied with CAM. Two thirds of these patients thought CAM was too expensive and the rest believed that CAM caused adverse effects. The same study reported exacerbated hypertension made up 74% of the adverse effects attributed by patients to CAM use [20] and all patients who reported adverse effects subsequently discontinued CAM use. 

### 3.6. Physician Awareness of Patients' Supplement Use

Six studies asked whether patients informed doctors about supplement use and found physicians were not notified 44% (IQR 40%–53%) of the time [6, 14, 15, 17, 20, 24]. One study reported that 67% of supplement users did not disclose supplement use because their doctors did not ask [14], but other studies did not investigate the reasons behind nondisclosure. 

## 4. Discussion

This is the first systematic review of dietary supplement use in cardiac patients and the findings have strong implications for clinical practice. The results indicate that supplement use is common in cardiac patients (26%–42%) and that the concomitant use of dietary supplements and prescription medicine also appears to be frequent (16%–64%). Sicker patients may be more likely to seek out alternative treatments [8, 13, 21], and drug interaction could play a causal role in the lower INR observed among supplement users on warfarin [22] or in the lower blood pressure among cardiac failure patients [9]. 

The potential for negative interaction in supplement users is high. Substances such as fish oil, hawthorn, garlic, ginseng, ginkgo, glucosamine, and parsley have antiplatelet properties [27, 28] and may interact with prescription antiplatelets or anticoagulants. Supplements such as capsicum and ginseng have been shown to affect blood pressure [29]. Supplemental potassium was taken by 1 in 5 respondents in one study [7]; this may result in adverse outcomes when used in combination with commonly prescribed cardiovascular medications such as angiotensin converting enzyme inhibitors, aldosterone receptor antagonists, or angiotensin receptor blockers. Other researchers have thoroughly reviewed the effects of herbal medicines and dietary supplements for cardiovascular health [30] as well as their potential for drug interaction [31] and we refer clinicians to these resources for more details. 

It is worth noting that patients are likely to be using more than one supplement at a time [21], for periods of months to years [13, 24]. This raises concerns of possible ongoing adverse interaction between supplements and prescription medications, as well as the potential for negative effects on compliance with conventional therapy. Many patients believe supplements are safe, effective, and produce few side effects. Many are also unaware that supplements can negatively impact prescription medications [14, 15, 24]. Moreover, a large proportion of physicians (39%–95%) are unaware of supplement use by their patients. Although patients may be reluctant to disclose supplement use, the main reason for nondisclosure appears to be physicians not asking about supplement use [14]. This suggests physicians can produce significant change in this aspect by being more proactive and asking about supplement use. 

There are inconsistent results with regard to the association between supplement use and compliance. There is also limited evidence on whether supplement use affects clinical outcomes such as blood pressure and cholesterol control. The available evidence suggests that supplement use is associated with differences in health status of cardiac patients, although it is unknown whether supplement use causes these differences or whether differences in health cause patients to self-manage through supplementation. 

The aggregated results presented here should be treated with caution. Our results show wide variability in the prevalence of dietary supplement use, which is consistent with other reviews of the CAM literature [32, 33]. Some of these variations may be due to the country and year in which the study was conducted. For the North American studies, the range of figures is smaller than for all studies but still large. For vitamin/mineral use it is 38% (IQR 26–49), for herbals 10% (IQR 7–18), and for other supplements 7% (IQR 5%–13%). In terms of variations over time, rates of supplement use seem to be decreasing over time, the overall rates being negatively correlated to the publication year in the North American studies (*r* = −0.30). However, there are also severe methodological limitations in the quality and comparability of the reviewed studies. For instance, even though many of the included studies involved clinical samples, few provided objective data on the health status of the patients. Clinical diagnoses of cardiovascular disease were reported in only seven studies [7, 9, 10, 14, 19, 23, 25], and only six compared supplement users and nonusers on clinical outcomes [7, 9, 10, 17, 21, 22]. Study comparison was also difficult due to heterogeneity in the definitions of dietary supplements and supplement use. For instance, some studies did not include common vitamins and supplements in their surveys, which would radically reduce the rates of supplement use. Some studies provided a checklist of supplements with no room for respondents to list other substances they may have used. Across studies, patients were asked whether they had used dietary supplements in the past year [6, 8, 12, 14, 16], in the past month [13], in the past 3 and 6 months [7, 17, 19, 25], as well as whether ever used in lifetime [15, 18]. Two studies defined regular use as “at least once weekly” [7, 19] and only one asked for the duration of use [24]. An obvious result of using dissimilar definitions are the disparate prevalence rates even in the largest population studies, all conducted in North America [6, 11, 13], which found supplement use to be 36%, 61%, and 22%, respectively. 

Future studies should focus on developing three main areas. The first is to standardise and make explicit the definitions of dietary supplements and supplement use, which will facilitate cross-study comparison [33]. The second is to examine the impact of supplement use on prescription medications. Our paper found inconsistent associations between supplement use and compliance with prescription medications. Given that long-term compliance is essential to cardiac care, investigation into whether supplement use disrupts compliance is warranted. This is particularly important in population groups susceptible to noncompliance. In addition, the reluctance of patients to disclose use of dietary supplements may require patient and physician education. Lastly, studies are needed to examine both health and financial costs of supplement use. Only one of the reviewed studies examined the relative cost of CAM, finding that patients of an Indian clinic were spending the same amount on CAM and antihypertensive treatment, although the authors note that the cost of mainstream care can be reimbursed in most cases whilst CAM treatment cannot [20]. Prospective studies can assess whether concomitant use of prescription medications and supplements in cardiac patients affects outcomes such as morbidity, mortality, and quality of life, while economic evaluation can determine whether supplement use is cost effective, especially given the wide availability, increasing accessibility, and now common use of dietary supplements.

In summary, the use of dietary supplements is common in patients with cardiovascular conditions. There are no conclusive findings with respect to the health factors associated with this use although many commonly used supplements have the potential to interfere with the intended action of prescription medications. A substantial number of treating physicians are unaware of patients' supplement use, and consequently the effects of supplementation on conventional treatment may be overlooked. Disclosure of supplement use can be facilitated if medical practitioners are more proactive in questioning patients.

## Figures and Tables

**Table 1:** Comparison of methodology in studies of dietary supplement use in cardiac patients.

Study	Dates of study	Geographic location	Mode of data collection	Validity & reliability of measure	Sample size	Response rate	Representativeness of sample	Definition of dietary supplements (nonsupplement CAM surveyed are not listed)	Definition of use
Ackman et al. (1999) [7]	July 1995–June 1996	Canada, Alberta	Patient survey	Piloted survey	180	75%	Respondents and nonrespondents comparable in age and gender	Vitamins/minerals, nutritional supplements, health food or herbal products	Used at least once weekly over last 3 months

Ai and Bolling (2002) [8]	May 1999–December 2000	US, Michigan	Face-to-face interview followed by telephone interview	—	225	48%	—	Herbal medicine, megavitamins, homeopathy, folk remedies	Use in last 1 year

Albert et al. (2009) [9]	April 2007–February 2008	US, Ohio & Pennsylvania	Telephone or face-to-face interview	Survey reviewed by research committee	374	—	—	Herbal therapies	—

Amira and Okubadejo (2007) [10]	3 month period	Nigeria, Lagos	Face-to-face interview	—	225	—	—	Dietary supplements and herbs	—

*Artz et al. (2006) [11]	Data from 2000-2002 Minnesota Heart Survey	US, Minnesota	Face-to-face interview	Validated interview, trained interviewers	315	—	Respondents compared to local census data	Nonvitamin/mineral dietary supplement, vitamin/mineral dietary supplement.	Use in last 2 weeks

Barraco et al. (2005) [12]	3 month period	US, Michigan	Face-to-face interview	Trained Interviewers	223	26%	—	Folk remedies, herbal therapy, homeopathy, megavitamin therapy (not daily vitamin), minerals (not calcium or iron), native American medicine, Tibetan medicine, traditional Chinese medicine	Use in last 12 months

*Buettner et al. (2007) [13]	Data from 1999-2000 and 2001-2002 National Health and Nutrition Examination Surveys	US	Face-to-face interview	—	1066 and 2482	82% and 84%	Representative respondents sampled	Vitamins, minerals, other dietary supplements	Use in last 1 month

Chagan et al. (2005) [14]	February 2001–December 2002	US, New York	Face-to-face interview	—	198	—	—	Herbal supplements, vitamins, mineral supplements	Use in last 12 months

Dal Corso et al. (2007) [15]	August-September 2005	Italy, Verona	Face-to-face interview	—	153	57%	—	Herbal remedies, integrators (vitamins, minerals, salts)	Ever used

Gohar et al. (2008) [16]	February–April 2006	UK, Birmingham	Face-to-face interview	—	153	79%	—	Alternative medical system, Vitamin supplements, dietary supplement, herbal medicine	Use in last 1 year

									
Krasuski et al. (2006) [17]	—	US, Texas	Patient survey	—	210	75%	—	Herbal medications, vitamin supplements (unless prescribed), dietary supplements	Use in last 6 months

Liu et al. (2000) [18]	March–May 1998	US, New York	Patient survey	—	263	70%	—	Chelation, herbs, homeopathy, naturopathy, nutritional therapy, vitamins	Ever used

Pharand et al. (2003) [19]	December 1998–February 1999	Canada, cross-country	Patient survey	Survey used in Ackman et al. (1999) [7]	306	—	Respondents distributed across country similarly to population	Vitamins or mineral products, nutritional supplements, health food or herbal products (home remedies, oriental remedies)	Used daily or at least once weekly in last 6 months

Shafiq et al. (2003) [20]	May 2001–October 2001	India, Chandigarh	Face-to-face interview	—	521	100%	—	Herbal medicine, homeopathy	Current and past use

Stys et al. (2004) [21]	—	US, New York	Physician interview	—	187	—	—	Vitamin, mineral or herbal supplements	Current (medication history)

Wong et al. (2003) [22]	May 2001	Hong Kong	Physician interview	—	107	100%	NA	Herbal decoctions, proprietary medicines containing herbs, “cool tea”, “herbal soup”, tonics	Use in last 1 week

Wood et al. (2003) [23]	—	Canada, Nova Scotia	Telephone interview	Trained interviewers	107	88%	Respondents younger (64 versus 69) but similar gender mix to nonrespondents	Megavitamins, herbal therapy, nutritional supplements, homeopathy, folk remedies, chelation	—

*Yeh et al. (2006) [6]	Data from 2002 National Health Interview Survey	US	Face-to-face interview	—	10572	74%	Representative respondents sampled	Alternative medical systems, chelation, folk medicine, herbal products, large-dose vitamins, special diets	Ever used, used in last 12 months

Yilmaz et al. (2007) [24]	—	Turkey	Face-to-face interview	Survey piloted	310	—	—	Herbals	Ever used, use daily, duration of use

Zick et al. (2005) [25]	January 2000–February 2004	US, Michigan	Patient survey	—	252	80%	—	Herbs, vitamins, minerals, amino acids, other	Use in last 6 months

Note: -: not reported, NA: not applicable, ∗Study analysed data from a population survey.

**Table 2:** Comparison of results in studies of dietary supplement use in cardiac patients.

Study	Setting	Clinical characteristics of sample	Mean age of sample (SD; range)	% female	Ethnic composition	Prevalence of supplement use	Associations between cardiovascular health, medication and supplement use	Reasons for use	Physician awareness
Ackman et al. (1999) [7]	Hospital clinic	Diagnosed congestive heart failure	69 (−)	37%	—	Vitamins/minerals 59% Nutritional supplements 17% Health food/herbal products 38%	Use unrelated to CHF severity	—	—

Ai and Bolling (2002) [8]	Cardiac clinic	Admitted for non-emergency/non-transplant surgery	62.4 (36–84)	44%	—	Megavitamins 13% Herbs and folk remedies 12% Homeopathy 4%	—	—	—

Albert et al. (2009) [9]	Hospitals and cardiology practices	Diagnosed heart failure	69.6 (±13.1; 31–98)	37%	81% Caucasian	Multivitamins/minerals 36% Herbal therapy 12% 60% users take with prescription medication	Users more likely than nonusers to be under cardiologist care, have high cholesterol, diabetes but not significantly different ejection fraction	Promote or maintain good health 45%–47% Overall wellbeing 30%–42% Energy/decrease fatigue 13%–25%	—

Amira and Okubadejo (2007) [10]	Hypertension clinic	Diagnosed hypertension	55.1 (±12.4)	60%	—	Dietary supplements and herbs 97%	Users had higher BMI (29 versus 27) but no different on blood pressure readings or duration of hypertension. Users no different on medication compliance and blood pressure control	—	—

Artz et al. (2006) [11]	Community	Taking nonprescription medication for cardiac reason	—	69%	—	Vitamin/mineral supplement 38% Nonvitamin/mineral supplement 21%	36% vitamin/mineral supplement users and 16% other supplement users also take prescription cardiac medication	—	—

Barraco et al. (2005) [12]	Hospital cardiology unit	Suspected acute coronary syndrome	66.2 (±13.4)	39%	79% Caucasian, 13% African-American	Herbals 5% Anti-oxidants 4% Minerals 3%	—	—	—

Buettner et al. (2007) [13]^#^	Community	Self-reported CAD/stroke or hypertension/high cholesterol	CAD/stroke 66 (−) hypertension/high cholesterol 58 (−)	46%/57%	80%/78% White, 10%/10% Black, 3%/4% Mexican-American	Multivitamin 36%–40% Minerals 9%–11% Herbs 7%–10% Other supplements 6%-7% Of cardiac medication users: Multivitamin 36%–40% Minerals 25%–30% Herbs 6%–9% Other supplements 6%-7%	Of those taking medication for cardiovascular disease, 63% of those with previous CAD/stroke and 64% of those with hypertension/high cholesterol also used a supplement CAD/stroke group more likely to use vitamin E, B vitamins and less likely to use fish oil; hypertension/high cholesterol group more likely to use garlic and ginseng	—	—

									
Chagan et al. (2005) [14]	Hospital clinic	Diagnosed cardiovascular disease	users 61.4 (±16.7) nonusers 58.7 (±16.3)	40%	50% White, 23% African-American, 15% Hispanic, 12% other	“Biological-based therapy”: ever used 48%, used in last 12 months 42%	No difference in users and nonusers in number of cardiovascular diseases. Average of 2 supplements and 7 prescription medications used per patient. 42 potential interactions identified, most commonly aspirin and vitamin E in 16 patients	18 of 42 supplements taken for a cardiac reason 75% users perceive safe, 70% effective, 45% believe cause fewer side effects than prescription medication	40% physicians unaware 67% of users were not asked by physicians about use

Dal Corso et al. (2007) [15]	Outpatient heart failure clinic	Clinic patients	65.7 (±10)	11%	—	Vitamins/minerals 21% Herbal medicine 9%	Only 1 patient from 153 reduced and interrupted heart failure tablets	—	39% did not report herbal use to doctors 44% did not report vitamin/mineral use

Gohar et al. (2008) [16]	Outpatient hypertension clinic	Clinic patients	57.3 (±16)	46%	64% White European, 25% South Asian, 11% Afro-Caribbean	Any biological-based therapy 29% Vitamin supplements 20% Dietary supplements 11% Herbal medicine 7%	—	—	—

Krasuski et al. (2006) [17]	Outpatient cardiology clinic	Returning clinic patients	66.2 (±11.6)	32%	—	Multivitamins 68%	No difference between users and nonusers on use of aspirin, betablockers, ACE-inhibitors, statins or Coumadin	Greater benefit than prescription 15%, safer than prescription 45%, no interactions 47%	50% did not inform cardiologists

Liu et al. (2000) [18]	Cardiothoracic surgery clinic	Patients attending clinic	41% aged 40–65, 51% aged over 65	28%	76% White, 7% African American, 6% Hispanic, 6% Native American	Vitamins 54% Herbs 10% Homeopathy 3%	—	—	—

Pharand et al. (2003) [19]	Hospital ward, outpatient clinic and emergency room	Diagnosed with CHF or CAD for at least 6 months	66 (40% over 70)	41%	—	Multivitamin/mineral 23% Single-entity vitamin/mineral 38% Herbal product 17%	—	—	—

Shafiq et al. (2003) [20]	Hospital hypertension clinic	Patients attending clinic	71% aged over 50, 26% aged 36–50, 3% aged 18–25	26%	—	Ayurveda 57% Herbal medicine 14% Homeopathy 8%	—	Adverse reactions of conventional therapy 59% Recommended by family/friends 62%	5% informed doctors about CAM

Stys et al. (2004) [21]	Preventive cardiology clinic	Patients attending clinic	61 (±14)	28%	—	Vitamins 53% Herbs 21% 1 or more supplements 57% Average of 3 supplements per user	Users more likely to have established CAD, family history of premature CAD, higher total cholesterol, higher HDL and use statins Users and nonusers have similar blood pressure and HbA1c levels	—	—

Wong et al. (2003) [22]	Warfarin clinic	Patients attending clinic	Users 56.5 (±12.4) Nonusers 56.4 (±11.7)	59%	—	Herbal medicine 26%	INR for users lower than for nonusers No difference between herbal users and nonusers in indication for Warfarin, duration of Warfarin therapy or Warfarin dosage	—	—

Wood et al. (2003) [23]	Registry of patients with cardiovascular disease	Previously hospitalised with ischaemic heart disease, CHF or AF	64.3 (±11.9)	35%	—	Megadose vitamins 35% Herbal supplements 32% Non-herbal supplements 22%	Of 24 patients taking warfarin, amiodarone, sotalol, or digoxin, 50% also used vitamin, 36% herbal, 36% non-herbal supplement	Potential improvement in condition 57%, proven benefit 28%, dissatisfied with conventional treatment 17%	—

Yeh et al. (2006) [6]	Community	Self-reported cardiovascular disease	35% aged 65 and over; 31% aged 50–64	53%	82% White, 13% Black, 5% other	High-dose vitamins 3% Herbal therapies 18%	Similar rates of vitamin and herbal use in those with and without cardiovascular disease	80% herbal users perceived herbs to be helpful	56% did not disclose herbal use

Yilmaz et al. (2007) [24]	Outpatient cardiology clinic	Admitted to clinic	males 54.7 (±13.3), females 56.2 (±14.7)	46%	—	Herbals 39% Of users: 55% used daily with average duration of 39 months (±70 months)	Users and nonusers were similar on diabetes, smoking, prior MI and prior PCI	19% used for hypertension, 23% for high cholesterol	92% did not inform physicians

Zick et al. (2005) [25]	Heart failure clinic	Diagnosed congestive heart failure	57 (±10)	33%	—	Any supplement 33%	82% used supplement for cardiac problem: 62% for CHF, 13% angina, 6% for hypertension		—

Note: —: not reported, NA: not applicable, ^#^Study examined CAD/stroke and hypertension/hypercholesterolaemia groups separately and 2 figures are presented, one for each group.

**Table 3:** Common supplements used by cardiac patients, pooled results from included studies.

Supplement	Number of studies	Pooled *n*	Pooled sample	Pooled prevalence (%)
aloe vera	2	15	423	3.6
Bilberry	2	4	439	1.0
Calcium	6	1,017	4,629	22.0
cayenne pepper/capsicum	2	26	486	5.3
chromium/chromium picolinate	3	11	679	1.6
coenzyme Q10	7	58	2,335	2.5
Dandelion	2	4	359	1.0
Echinacea	5	705	11,503	6.1
fish oil/omega-3	7	279	14,014	2.0
flax seed/flax seed oil	5	48	1,235	3.9
folic acid	7	160	5,108	3.1
Garlic	12	902	16,417	5.5
Ginger	6	302	11,909	2.5
ginkgo biloba	6	572	15,131	3.8
Ginseng	8	502	14,471	3.5
glucosamine/chondroitin	8	499	11,855	4.2
green tea	4	61	931	6.6
Hawthorn	4	13	913	1.4
Iron	4	26	673	3.9
Kelp	2	7	486	1.4
Lecithin	3	8	492	1.6
multivitamins and minerals	9	1,883	5,525	34.1
Magnesium	6	50	1,352	3.7
mint/lemon balm	2	44	417	10.7
Nettle	3	68	864	7.9
Parsley	4	214	10,824	2.0
Peppermint	2	214	10,824	2.0
Potassium	3	87	4,034	2.2
Sage	2	29	417	7.0
saw palmetto	4	32	1,023	3.1
Selenium	2	8	385	2.0
St John's wort	3	220	11,053	2.0
Valerian	3	24	882	2.7
vitamin A/beta-carotene	6	107	4,671	2.3
vitamin B/B12/B complex	8	470	5,261	8.9
vitamin C	9	768	5,408	14.2
vitamin D	4	42	1,047	4.0
vitamin E	8	1,056	5,318	19.9
Zinc	5	37	1,123	3.3

Note: Only supplements with reported ≥1% use in 2 or more studies were included. Some figures were calculated from reported percentages.
